# Comparing the test–retest reliability of resting‐state functional magnetic resonance imaging metrics across single band and multiband acquisitions in the context of healthy aging

**DOI:** 10.1002/hbm.26180

**Published:** 2022-12-22

**Authors:** Marie‐Stephanie Cahart, Owen O'Daly, Vincent Giampietro, Maarten Timmers, Johannes Streffer, Steven Einstein, Fernando Zelaya, Flavio Dell'Acqua, Steven C. R. Williams

**Affiliations:** ^1^ Neuroimaging Department Institute of Psychiatry, Psychology and Neuroscience, Kings College London London UK; ^2^ Division of Janssen Pharmaceutica NV Janssen Research and Development Beerse Belgium; ^3^ AC Immune SA Lausanne Switzerland; ^4^ Reference Center for Biological Markers of Dementia (BIODEM) University of Antwerp Antwerp Belgium; ^5^ UCB Biopharma SPRL Brussels Belgium; ^6^ Natbrainlab; Forensic and Neurodevelopmental Sciences Department Institute of Psychiatry, Psychology and Neuroscience, Kings College London London UK

**Keywords:** connectivity, fMRI, multiband fMRI, reliability, resting‐state

## Abstract

The identification of meaningful functional magnetic resonance imaging (fMRI) biomarkers requires measures that reliably capture brain performance across different subjects and over multiple scanning sessions. Recent developments in fMRI acquisition, such as the introduction of multiband (MB) protocols and in‐plane acceleration, allow for increased scanning speed and improved temporal resolution. However, they may also lead to reduced temporal signal to noise ratio and increased signal leakage between simultaneously excited slices. These methods have been adopted in several scanning modalities including diffusion weighted imaging and fMRI. To our knowledge, no study has formally compared the reliability of the same resting‐state fMRI (rs‐fMRI) metrics (amplitude of low‐frequency fluctuations; seed‐to‐voxel and region of interest [ROI]‐to‐ROI connectivity) across conventional single‐band fMRI and different MB acquisitions, with and without in‐plane acceleration, across three sessions. In this study, 24 healthy older adults were scanned over three visits, on weeks 0, 1, and 4, and, on each occasion, underwent a conventional single band rs‐fMRI scan and three different rs‐fMRI scans with MB factors 4 and 6, with and without in‐plane acceleration. Across all three rs‐fMRI metrics, the reliability scores were highest with MB factor 4 with no in‐plane acceleration for cortical areas and with conventional single band for subcortical areas. Recommendations for future research studies are discussed.

## Graphical abstract

We compared the test–retest reliability of three resting‐state fMRI metrics across four different resting‐state modalities and over three visits. Multiband factor 4 with no in‐plane acceleration significantly improved intraclass correlation coefficient (ICC) scores for cortical areas, while single band yielded the best ICC values for subcortical regions. We recommend multiband factor 4 with no in‐plane acceleration for whole brain analyses or analyses focussing specifically cortical regions, and single band for studies with an emphasis on subcortical areas.

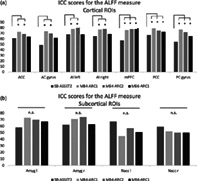

## 1 INTRODUCTION

Over the past 20 years, resting‐state functional magnetic resonance imaging (rs‐fMRI) research has proven invaluable for shedding light on intrinsic functional networks in the brain at rest (Fox & Raichle, 2007). More specifically, studies have suggested that rs‐fMRI may be used to explore the neural architecture of developmental, aging, and pathological processes and help establish predictive biomarkers for mental illness (Fox & Raichle, 2007; Greicius, 2008). Identifying robust and trustworthy biomarkers necessitates measures that have satisfactory test–retest reliability over multiple visits. Indeed, reliable measures are one of the cornerstones of scientific progress as they ensure that similar results are observed when the same scan sequence is repeated on the same group of subjects over several visits.

In the last two decades, concerns regarding the reliability of biomedical research have been increasingly expressed (Ionnadis, 2005; Prinz et al., 2011) and recent research has focused on identifying best practices with a view to improving the test–retest reliability of standard rs‐fMRI measures (Noble et al., 2019; Zuo & Xing, 2014). Overall, the observed test–retest reliability scores of rs‐fMRI metrics varies greatly across studies, with reliability scores ranging between poor (Noble et al., 2019; Shou et al., 2013) and moderate to excellent (Birn et al., 2013; Guo et al., 2012; O'Connor et al., 2017). These discrepancies across studies can be attributed to distinct factors that have previously been highlighted as having a direct impact on test–retest reliability. Indeed, recent literature has emphasized that reliability tends to be higher for brain regions located in the cortex rather than the subcortex (Shah et al., 2016) and for measures focusing on the regional intensity of spontaneous brain activity, also known as amplitude of low‐frequency fluctuations (ALFF; Wang et al., 2013) compared with measures of functional connectivity (FC) per se such as seed‐to‐voxel activation maps (Shou et al., 2013). Nonetheless, while the literature points towards these factors as having a major impact on test–retest reliability, other features remain to be explored such as those associated to recent developments in magnetic resonance physics.

Echo planar imaging (EPI) was first introduced by Mansfield (1977) and, since the early 1990s, has been mostly used for blood oxygen level‐dependent (BOLD) fMRI and diffusion weighted imaging investigations. Whilst conventional EPI can acquire a whole brain image in 2–3 s, multiband (MB) acquisitions, first introduced by Larkman et al. (2001), simultaneously excite multiple slice locations, thus decreasing the time needed to scan a whole brain volume to <1 s (Liao et al., 2013). However, imperfections in the excitation profile of the MB RF pulses can lead to (1) signal leakage between simultaneously excited slices (Todd et al., 2016); (2) signal dropout resulting in decreased temporal signal to noise ratio (tSNR; Chen et al., 2015); and (3) enhanced spatially heterogeneous noise amplification which increases at higher MB factors (Risk et al., 2021).

To overcome issues related to signal dropouts and susceptibility‐related distortions, in‐plane acceleration has commonly been added in contemporary MB fMRI studies. A recent study has shown that a total acceleration of 4 (i.e., MB factor 2 with in‐plane acceleration 2) is optimal with regards to sensitively detecting common rs networks while offering a negligible decrease in signal to noise ratio compared with a total acceleration of 2, 6, and 8 (Preibisch et al., 2015). Other studies have recommended the use of a MB factor of 4 for whole‐brain fMRI scanning, while single band acquisitions are reported to be better suited for studies focused on activity in subcortical regions due to differences in signal detection sensitivity (Risk et al., 2021).

In terms of test–retest reliability, however, only a few studies have compared the impact of different TRs and acceleration factors on the reliability of commonly used rs‐fMRI metrics (Golestani et al., 2017; Wang et al., 2013). Both studies showed the reliability of the ALFF measure to be higher with shorter TRs compared with a conventional rs‐fMRI sequence with a lower sampling rate (Golestani et al., 2017; Wang et al., 2013). Limitations of these studies include a small sample size (*n* = 8; Golestani et al., 2017), a comparison based solely on sampling rate rather than a clear comparison between MB and single band sequences (Golestani et al., 2017) and the comparison of data acquired from different scanners (Wang et al., 2013). To our knowledge, no studies have yet explored which combination of MB factor and in‐plane acceleration yields the best test–retest reliability in comparison with single band sequences all acquired from the same scanner and with a reasonable sample size in the context of three different rs‐fMRI measures such as ALFF, seed‐to‐voxel analysis and region of interest (ROI)‐to‐ROI analysis. In this study, we aim to address this need.

Based on the studies cited above, particularly those that show altered sensitivity subcortically, we hypothesized that (1) reliability scores would be significantly higher for MB protocols compared with single band for cortical regions, while single band would be best suited for subcortical regions. More specifically, we also hypothesized that, (2) for cortical regions, MB4 with no in‐plane acceleration (i.e., a total acceleration of 4) would yield the best reliability scores, while MB4 with in‐plane acceleration 2 (i.e., a total acceleration of 8) would be the MB modality associated with the lowest reliability scores. We also hypothesized that (3) reliability scores would be higher with the ALFF measure compared with the seed‐to‐voxel metrics, and (4) would be higher for cortical regions compared with subcortical regions across all three metrics.

## 2 MATERIALS AND METHODS

### 2.1 Participants

In total, 30 healthy right‐handed adults aged 52–73 (19 males and 11 females) participated in the study after providing written informed consent. All participants met the following inclusion criteria: being right‐handed, aged between 50 and 75, being physically healthy, not having any MRI counter‐indications (i.e., pacemaker, heart valve, metal in the body, claustrophobia), not suffering from any psychiatric or neurological disorder and not being on any psychoactive medication. Of the 30 participants who took part, two dropped out before completing all three scans and the data from four further participants were discarded due to technical issues that occurred during the scans. Therefore, the final number of participants included in the analyses was 24 (15 males and 9 females, age = 61.3 ± 7.9 years). The study was approved by the King's College London human Research Ethics Committee (number HR‐17/18‐5720). After each visit, the researchers visually inspected the scans for artifacts and all scans were reviewed by a qualified radiologist in order to rule out any neurological disorder, in line with the Department of Neuroimaging's standard policies. Unprocessed EPI images of slice 21 in axial view for run 1 for each participant and each rs‐fMRI modality can be found in Figure S1.

None of the participants had any history of psychiatric disorder or neurological disease or received any psychoactive treatment during the study.

### 2.2 Procedure

Each participant was invited to attend three scanning sessions at the Centre for Neuroimaging Sciences (Institute of Psychiatry, Psychology and Neuroscience; King's College London), on Weeks 0, 1, and 4 (±1 day), at the same time of day (±1 h).

### 2.3 MRI data acquisition

On each of the three visits, all participants were scanned in the same 3 T MRI scanner (Discovery MR750, General Electric, Milwaukee, Wisconsin). All of the images were acquired by experienced qualified radiographers who all rigorously followed the exact same MRI protocol. All participants underwent an anatomical T1‐weighted MRI and four rs sequences: (1) standard single band Echo‐Planar Imaging, with an in‐plane acceleration of 2 (SB‐ASSET = 2); (2) MB4, with no in‐plane acceleration (MB = 4, ARC = 1); (3) MB4, with an in‐plane acceleration of 2 (MB = 4, ARC = 2); (4) and MB6, with no in‐plane acceleration (MB = 6, ARC = 1). ASSET, also known as “Array Coil Spatial Sensitivity Encoding,” corresponds to the methodology which we employed for the single band sequence and which consists of parallel imaging with in‐plane acceleration, combining data in image space as it is commonly done when using “Sensitivity Encoding” or “SENSE” acceleration. For MB sequences, we used ARC, also known as “Auto‐calibrating Reconstruction for Cartesian Imaging,” which corresponds to parallel imaging with data combination in k‐space, as also performed by “Generalized Auto‐calibrating Partial Parallel Acquisition.” For this study, we used the Nova 32‐channel head coil.

The acquisition parameters for each rs‐fMRI sequence are described in Table 1. The order of the four rs‐fMRI runs was counterbalanced across imaging sessions and subjects. The anatomical sequence had the following parameters: repetition time = 8.23 ms; echo time = 3.25 ms; flip angle = 12°; field of view = 230 mm^2^; matrix size = 256 × 256; 1 mm isotropic resolution.

**TABLE 1:** Parameters of resting‐state fMRI sequences

	Repetition time (ms)	Echo time (ms)	Flip angle (°)	Field of view (mm^2^)	Matrix size	Time points
SB‐ASSET2	2000	30	82	211	64 × 64	240
MB4‐ARC1	750	30	63	211	64 × 64	644
MB4‐ARC2	750	30	63	211	64 × 64	645
MB6‐ARC1	550	30	57	211	64 × 64	873

Abbreviation: fMRI, functional magnetic resonance imaging.

During the acquisition of all four rs‐fMRI sequences, the participants were asked to keep their eyes open and fixate on a cross‐presented on the screen. Additionally, they were provided with headphones and earplugs to reduce any discomfort associated with the noise of the scanner. Each of the four rs‐fMRI sequences was 8‐min long, with a higher number of images being collected as the MB factor increased as displayed in Table 1.

During the same data acquisition, a T2 FLAIR, a T2 CUBE, and three Diffusion Tensor Imaging modalities were also collected. However, we did not use them for this study.

### 2.4 MRI data preprocessing

The data were preprocessed using the Statistical Parametric Mapping (SPM12) software and the CONN toolbox Version 18b (Whitfield‐Gabrieli & Nieto‐Castanon, 2012). Preprocessing of the functional data included realignment (motion correction), registration to the structural image, spatial normalization into the Montreal Neurological Institute (MNI) standardized space and smoothing with a Gaussian filter of 5.0 mm spatial full width at half‐maximum value. Slice‐timing correction was carried out on the single band echo‐planar imaging sequence only due to slice timing effects being considerably larger for single band sequences compared with MB given the shorter repetition times and several slices being acquired at the same time. Physiological and other spurious sources of noise were estimated and regressed out using the aCompcor method (anatomical component‐based noise correction method; Behzadi et al., 2007) which performs principal components analysis to identify, for each subject, contributions from the white matter and the cerebrospinal fluid that are unlikely to derive from neural activity. A conventional bandpass filter over a low‐frequency window of interest (0.008–0.09 Hz) was finally applied to the rs time series across all four rs‐fMRI modalities.

### 2.5 tSNR analyses

In order to explore how tSNR differs across all four rs‐fMRI sequences in cortical regions and in subcortical regions, voxelwise whole‐brain tSNR maps were extracted for each participant, each run and each rs‐fMRI modality The tSNR maps were calculated using fMRI images prior to denoizing and before the data was demeaned. We then used a mask to spatially constrain each tSNR map to each of the 11 ROIs. The resulting maps were then averaged across all three runs and divided into two groups: a cortical group made up of all seven cortical ROIs and a subcortical group comprising all four subcortical ROIs. Within each group, global tSNR was assessed by averaging all the tSNR values across voxels and across all seven ROIs for the cortical group and all four ROIs for the subcortical group. The resulting averaged tSNR values were then submitted to paired *t*‐tests for each group. False discovery rate (FDR) correction (Benjamini & Hochberg, 1995) was used for adjusting for all three contrasts (i.e., SB‐ASSET2 compared with MB4‐ARC1; SB‐ASSET2 compared with MB4‐ARC2 and SB‐ASSET2 compared with MB6‐ARC1).

### 2.6 fMRI analyses

For all three analyses described below, we specifically focused on three rs‐fMRI metrics which have previously been used to gain a better understanding of psychiatric disorders: ALFF, seed‐to‐voxel and ROI‐to‐ROI measures (Ebisch et al., 2011, Lei et al., 2017; Yang et al., 2021, Zhang et al., 2021). All fMRI analyses were carried out using MR images after denoizing.

**FIGURE 1:**
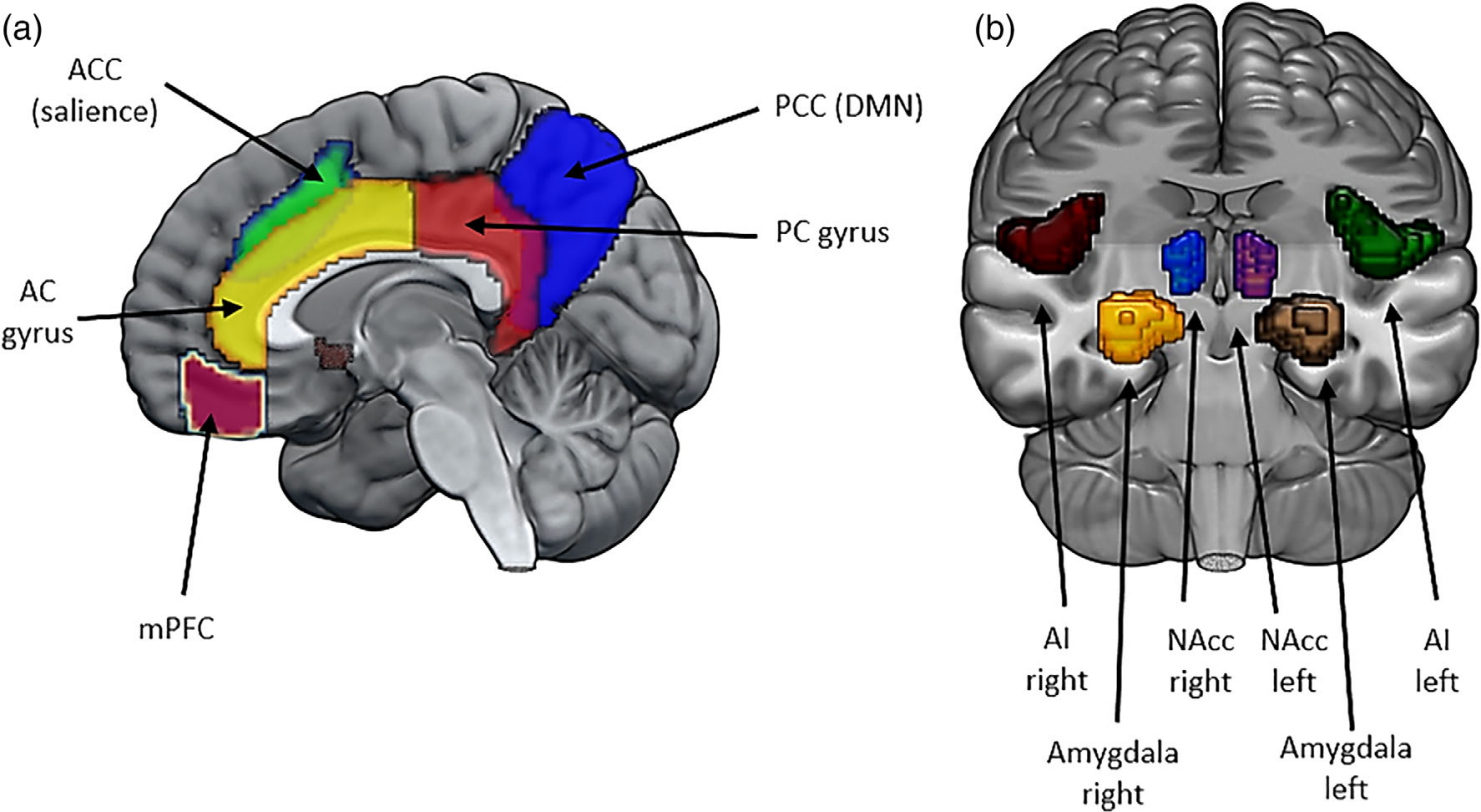
MRIcroGL (https://www.nitrc.org/projects/mricrogl/) was used to create overlay images representing all 11 seeds: Sagittal view (a) and coronal view (b). AC, anterior cingulate; ACC, anterior cingulate cortex; AI, anterior Insula, NAcc, nucleus accumbens; PC, posterior cingulate; PCC, posterior cingulate cortex

Eleven ROIs were identified a priori based on their implication in a range of psychiatric and neurological disorders (Ebisch et al., 2011; Ellard et al., 2018; Lei et al., 2017; Liu et al., 2021; Uddin et al., 2013; Wu et al., 2021; Yang et al., 2021; see Bao et al., 2021, Briley et al., 2022 and Wang et al., 2020 for meta‐analyses focusing on posttraumatic stress disorder, anxiety and depression, and bipoler disorder, respectively). The ROI name, MNI coordinates of the center of gravity of each ROI and rendering of all 11 ROIs are provided in Table 2 and Figure 1.

**TABLE 2:** ROI name and MNI coordinates of each of the 11 ROIs chosen a priori

ROI name	MNI coordinates
ACC, salience	0; 22; 34
AC gyrus	0; 18; 24
AI left, salience	−43; 12; 0
I right, salience	46; 14; 0
Amygdala left	−22; −4; −17
Amygdala right	23; −3; −17
mPFC, DMN	1; 55; −3
NAcc left	−9; 11; −7
NAcc right	9; 12; −6
PCC, DMN	0; −60; 38
PC gyrus	0; −36; 29

Abbreviations: AC, anterior cingulate; ACC, Anterior cingulate cortex; AI, anterior Insula; DMN, default‐mode network; MNI, Montreal Neurological Institute; mPFC, medial prefrontal cortex; NAcc, nucleus accumbens; PC, posterior cingulate; PCC, posterior cingulate cortex; ROI, region of interest.

It is worth noting that the anterior cingulate gyrus, the bilateral amygdalae, the bilateral nuclei accumbens and the posterior cingulate gyrus were anatomically defined, based on the Harvard‐Oxford cortical atlas implemented in the CONN toolbox (Whitfield‐Gabrieli & Nieto‐Castanon, 2012). In contrast, the other ROIs were functionally defined based on CONN's Independent‐Component Analysis of the HCP dataset (407 subjects; Whitfield‐Gabrieli & Nieto‐Castanon, 2012). More specifically, the ROIs referred to as anterior cingulate cortex and anterior insulae left and right represent key nodes of the salience network, whereas the ROIs referred to as medial prefrontal cortex and the posterior cingulate cortex represent major nodes of the default‐mode network (DMN) as described in Table 2. It is worth noting that the salience network and the DMN have previously been implicated in neurological and psychiatric disorders such as autism spectrum disorders (Ebisch et al., 2011), Alzheimer Disease (Zheng et al., 2019), bipolar disorder (Lei et al., 2017), major depression (Wu et al., 2021) as well as schizophrenia and obsessive–compulsive disorder (Zhang et al., 2021).

### 2.7 Intraclass correlation coefficient analysis

The intraclass correlation coefficient (ICC; Landis & Koch, 1977) was used to index the reliability of all three rs‐fMRI metrics described below. The ICC score is typically defined as the proportion of within‐subject variability in relation to between‐subject variability as follows, where MSE_b_ and MSE_w_ are the between‐subject and within‐subject mean squared errors, respectively (Charman et al., 2017):

$$\mathrm{ICC}=\frac{\mathrm{MS}E_{b}^{\prime \prime }-\mathrm{MS}E_{w}^{\prime \prime }}{\mathrm{MS}E_{b}^{\prime \prime }+\mathrm{MS}E_{w}^{\prime \prime }}$$

As such, the higher the ICC score, the more similar within‐subject measurements are over time. ICC scores typically range between −1 and 1; however, in this article, these values are scaled to a range of −100 and 100 and, in this case, ICC scores are categorized as poor (ICC < 21), fair (20 < ICC < 41), moderate (40 < ICC < 61), substantial (60 < ICC < 81) and almost perfect (ICC > 80; Landis & Koch, 1977).

For all analyses, the reliability scores were calculated across all three visits using the ICC toolbox (Caceres et al., 2009), a MATLAB toolbox designed specifically for voxel‐wise ICC analyses of neuroimaging data. ICC analyses were implemented voxel‐wise and ICC scores were derived from the median of the full distribution of the ICC values across all voxels in each brain region, a method which has previously been shown to be stable under different conditions of smoothing and cluster size (Caceres et al., 2009). In order to formally compare ICC scores across all four rs‐fMRI modalities, *F*‐tests were run for each ROI, testing the null hypothesis that the ICC value of a given rs‐fMRI modality was equal to that of another rs‐fMRI modality, in line with previous work by McGraw and Wong (1996). FDR (Benjamini & Hochberg, 1995) was applied to adjust for all 11 ROIs and all 6 contrasts (i.e., SB‐ASSET2 compared with MB4‐ARC1, MB4‐ARC2, and MB6‐ARC1; MB4‐404 ARC1 compared with MB4‐ARC2 and MB6‐ARC1; and MB4‐ARC2 compared with MB6‐ARC1).

### 2.8 ALFF analysis

The intensity of the brain's spontaneous activity can be examined through the ALFF measure which has previously been used as a marker for brain diseases (Cheng et al., 2013; Zang et al., 2007). The first step of the ALFF analysis consisted of transforming the timeseries to the frequency domain for each voxel using a fast Fourier transform, and then obtaining the power spectrum. Because ALFF is defined as the averaged square root of the amplitude within a specific frequency range (Zang et al., 2007), we calculated the ALFF by computing the average square root across 0.008–0.09 Hz for each voxel. Each ALFF map was then normalized with respect to the global mean ALFF value for standardization purposes as described in Zang et al. (2007). An ALFF map was then generated for each subject and each visit and the median ICC was calculated over all three visits.

### 2.9 Seed‐to‐voxel analyses

For each subject and each visit, we extracted the mean BOLD timeseries from each of the 11 seeds and calculated the Pearson's correlation coefficient between the timeseries of each seed and the timeseries of all other voxels in the brain. Correlation coefficients were then converted to normalized z‐scores using Fisher's transform. Eleven normalized correlations maps were thus obtained for each subject and for each visit.

For these analyses, the whole‐brain median ICC scores were first calculated across all voxels for each of the 11 correlation maps. We will refer to these analyses as seed‐to‐voxel in the rest of the article. Further ICC analyses were subsequently completed and consisted in using a mask to spatially constrain each of the 11 correlation maps to each of the 10 remaining ROIs. The median voxel‐wise ICC value was calculated for each ROI. From here on, we will refer to these spatially constrained ICC analyses as ROI‐to‐ROI.

## 3 RESULTS

### 3.1 tSNR analyses

Paired *t*‐tests revealed a significant difference in tSNR values in cortical areas between single band and MB6‐ARC1 [*t*_(23)_ = 3.291, *p* = .003], but not with the other two MB modalities. In subcortical areas, significant differences in tSNR were observed for SB‐ASSET2 compared with MB4‐ARC2 [*t*_(23)_ = 2.211, *p* = .037] and MB6‐ARC1 [*t*_(23)_ = 6.830, *p* < 0.001]. Full details about descriptive statistics and paired *t*‐tests are available in Table 3.

**TABLE 3:** Descriptive statistics and paired *t*‐tests for the tSNR measure before denoizing (*n*, number of participants; SD, standard deviation; SE, standard error)

	*n*	Mean	SD	SE	Paired t‐tests
Cortical ‐ SB‐ASSET2	24	136.91	34.80	7.10	*t*_(23)_ = −0.376, *p* = .710
Cortical—MB4‐ARC1		139.05	34.52	7.05	
Cortical—SB‐ASSET2	24	136.91	34.80	7.10	*t*_(23)_ = 0.323, *p* = .750
Cortical—MB4‐ARC2		134.01	33.71	6.88	
Cortical—SB‐ASSET2	24	136.91	34.80	7.10	*t*_(23)_ = 3.291, *p* = .003
Cortical—MB6‐ARC1		114.35	21.61	4.41	
Subcortical—SB‐ASSET2	24	129.25	35.85	7.32	*t*_(23)_ = 0.165 *p* = .871
Subcortical—MB4‐ARC1		128.41	27.21	5.55	
Subcortical—SB‐ASSET2	24	129.25	35.85	7.32	*t*_(23)_ = 2.211, *p* = .037
Subcortical—MB4‐ARC2		109.71	26.55	5.42	
Subcortical—SB‐ASSET2	24	129.25	35.85	7.32	*t*_(23)_ = 6.830, *p* < .001
Subcortical—MB6‐ARC1		80.70	12.85	2.62	

Abbreviation: tSNR, temporal signal to noise ratio.

### 3.2 ICC analyses

**FIGURE 2:**
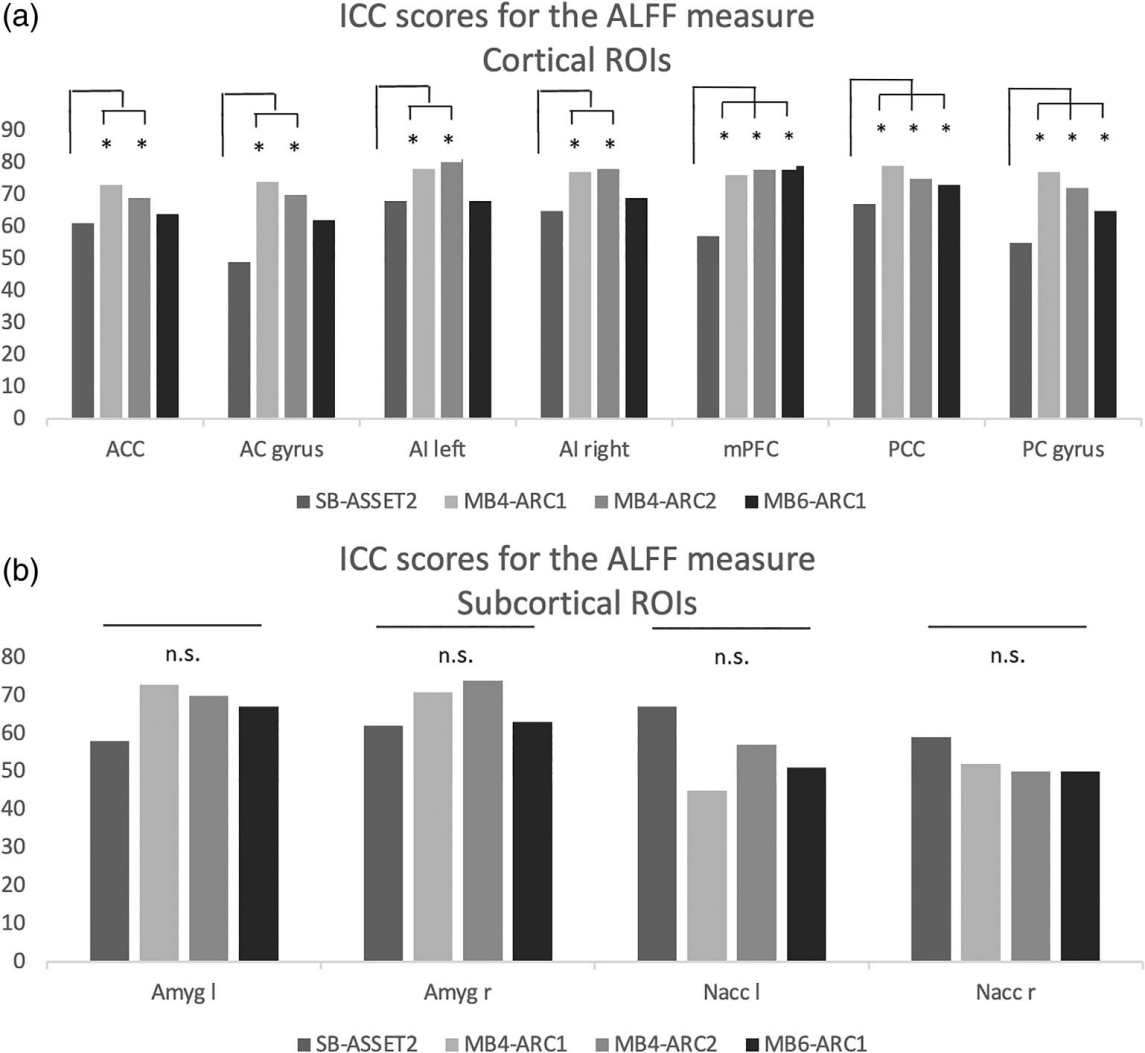
Formal comparison of the intraclass correlation coefficient (ICC) scores for the amplitude of low‐frequency fluctuations (ALFF) measure for each of the four resting‐state functional magnetic resonance imaging modalities, (a) for each of the cortical regions of interest (ROIs) and (b) each of the subcortical ROIs. *, significant *F*‐test, FDR‐corrected; AC, anterior cingulate; ACC, anterior cingulate cortex; AI, anterior Insula; DMN, default‐mode network; mPFC, medial prefrontal cortex; n.s., nonsignificant; PC, posterior cingulate; PCC, posterior cingulate cortex

**FIGURE 3:**
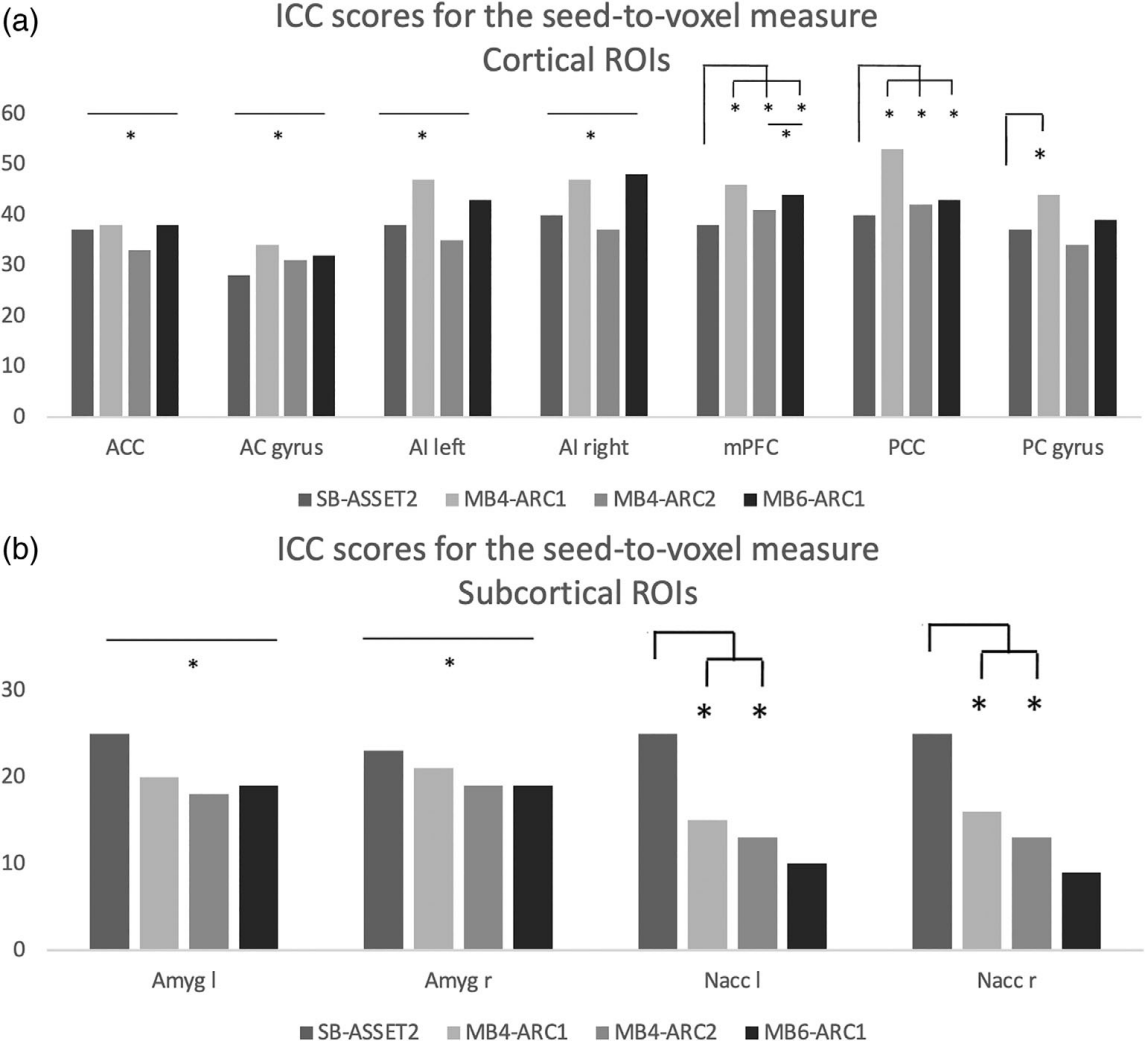
Formal comparison of the intraclass correlation coefficient (ICC) scores for the seed‐to‐voxel measure for each of the four resting‐state functional magnetic resonance imaging modalities, (a) for each of the cortical regions of interest (ROIs) and (b) each of the subcortical ROIs. *, significant *F*‐test, FDR‐corrected

**FIGURE 4:**
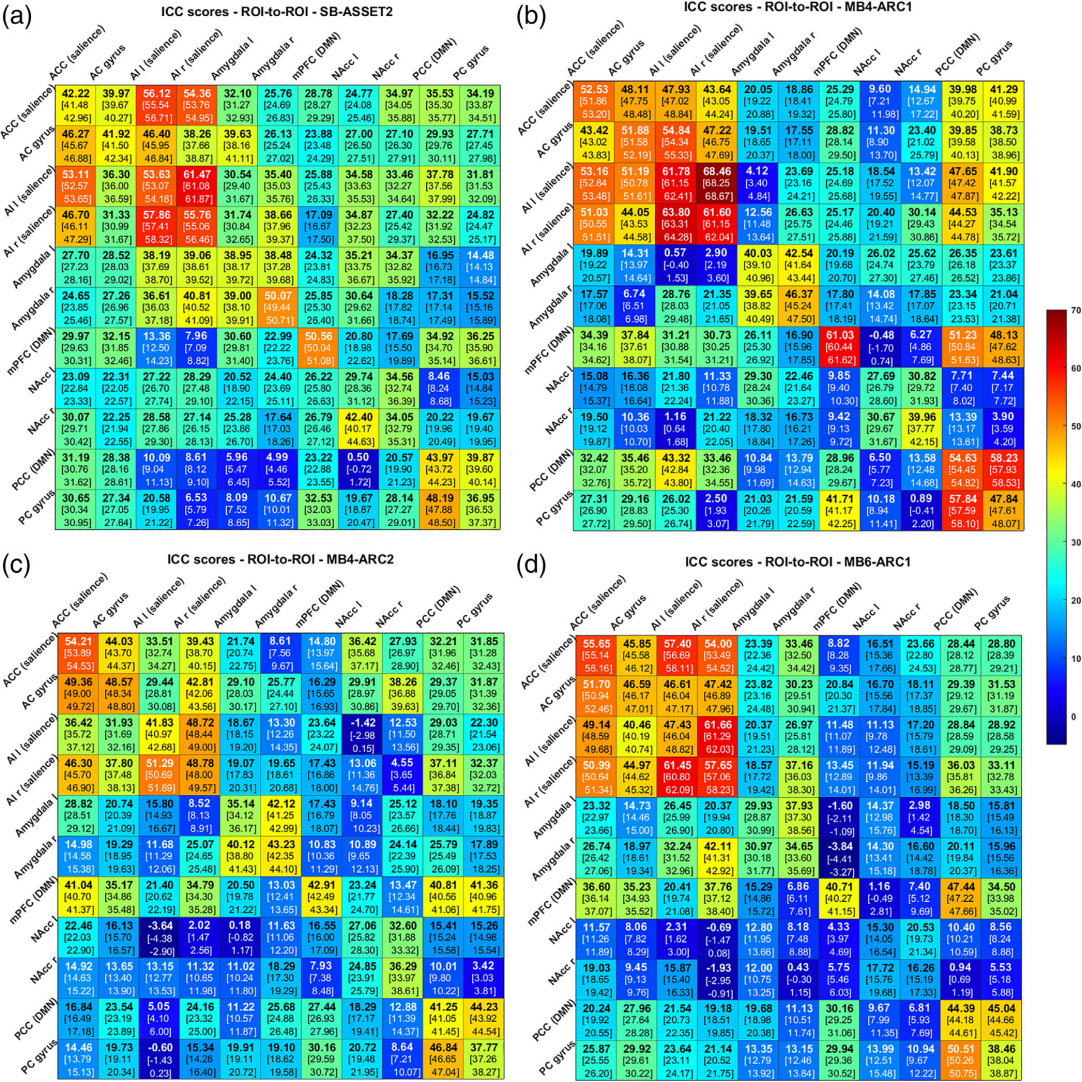
Intraclass correlation coefficient (ICC) scores [lower bound of the 95% confidence interval – Upper bound of the 95% confidence interval] for the region of interest (ROI)‐to‐ROI measure across all pairs of ROIs for SB‐ASSET2 (a), MB4‐ARC1 (b), MB4‐ARC2 (c) and MB6‐ARC1 (d). Warmer colors (i.e., red) represent higher ICC values, while colder colors (i.e., blue) represent lower ICC scores

**FIGURE 5:**
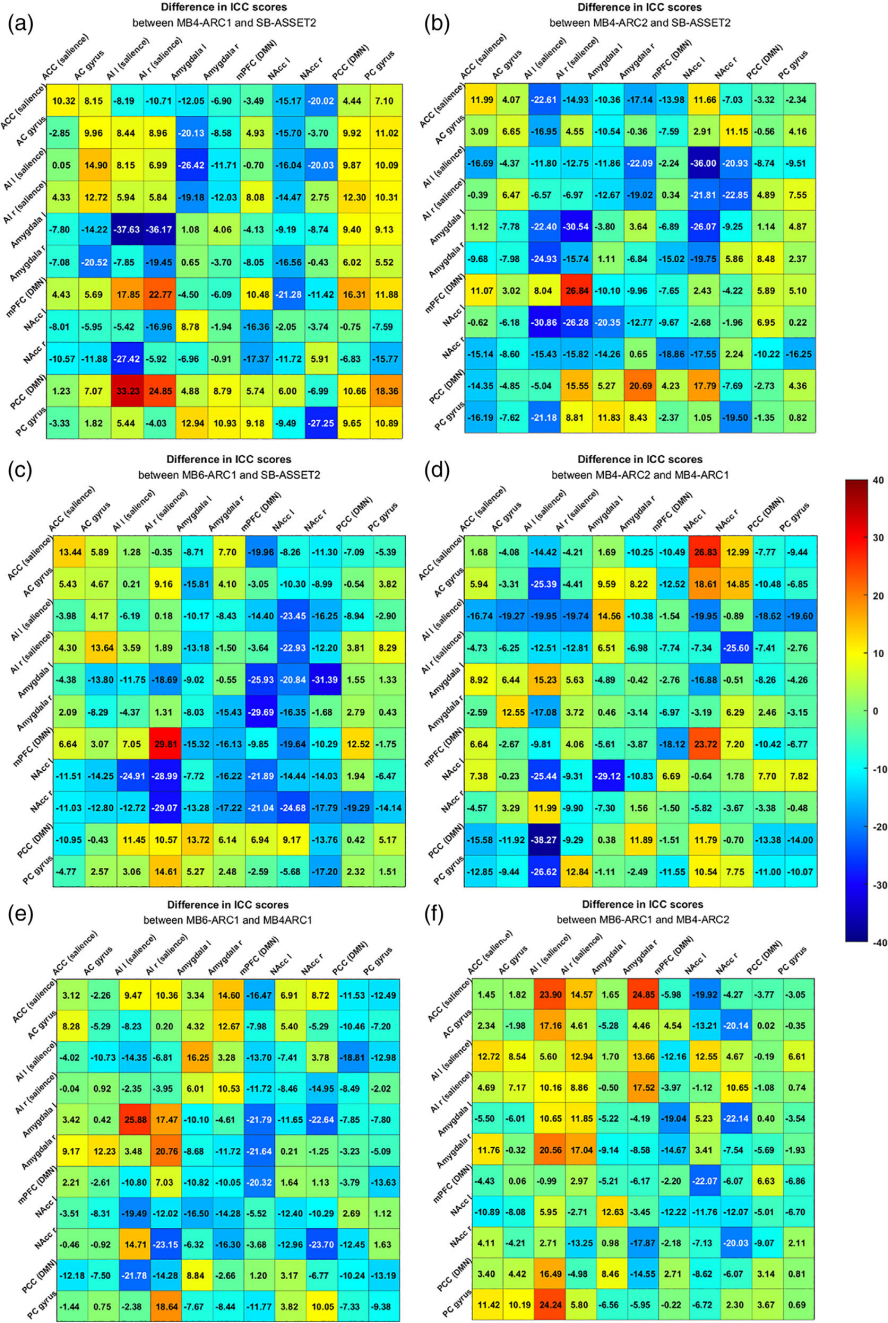
Differences in Intraclass correlation coefficient (ICC) scores between (a) MB4‐ARC1 and SB‐ASSET2, (b) MB4‐ARC2 and SB‐ASSET2, (c) MB6‐ARC1 and SB‐ASSET2, (d) MB4‐ARC2 and MB4‐ARC1, (e) MB6‐ARC1 and MB4‐ARC1, and (f) MB6‐ARC1 and MB4‐ARC2

In the results that follow, the observed ICC scores varied across rs‐fMRI modalities, rs‐fMRI metrics and brain regions (Figures 2, 3, 4, 5).

### 3.3 Test–retest reliability of the ALFF measure

Figure 2 displays the ICC scores for the ALFF measure for each of the 11 seeds across all four rs‐fMRI modalities. For cortical regions, the *F*‐tests revealed significantly higher ICC scores for all three MB sequences compared with the single band protocol for the medial prefrontal cortex, the posterior cingulate cortex and the posterior cingulate gyrus. For the anterior cingulate cortex, the anterior cingulate gyrus and the bilateral anterior insula, only MB4‐ARC2 and MB4‐ARC1 exhibited significantly higher scores compared with SB‐ASSET2. In addition, for the bilateral nuclei accumbens, scores were the highest for the single band modality but did not survive FDR correction. Full details about the ICC scores and the associated confidence intervals for the ALFF measure can be found in Table S1.

### 3.4 Test–retest reliability of the seed‐to‐voxel measure

Figure 3 displays the ICC scores for the seed‐to‐voxel measure for each of the 11 seeds across all four rs‐fMRI modalities. For the anterior cingulate cortex, the anterior cingulate gyrus and the bilateral anterior insulas, *F*‐tests revealed significant differences for each of the six contrasts (i.e., SB‐ASSET2 compared with MB4‐ARC1, MB4‐ARC2 and MB6‐ARC1; MB4‐404 ARC1 compared with MB4‐ARC2 and MB6‐ARC1; and MB4‐ARC2 compared with MB6‐ARC1). In particular, MB4‐ARC1 and M6‐ARC1 consistently exhibited the highest ICC scores compared with single band and MB4‐ARC2. For the medial prefrontal cortex, the posterior cingulate cortex and the posterior cingulate gyrus, scores were consistently the highest for MB4‐ARC1. In contrast, for subcortical regions (i.e., bilateral amygdalae and nuclei accumbens), scores were the highest with single band and then decreased with increasing MB factors. Full details about the ICC scores and the associated confidence intervals for the ALFF measure can be found in Table S2.

### 3.5 Test–retest reliability of the ROI‐to‐ROI measure

Figure 4a–d displays the ICC scores for the ROI‐to‐ROI measure for each of the pairs of ROIs across all four rs‐fMRI modalities. Highest ICC scores were obtained with MB4‐ARC1 for pairs of ROIs involving two cortical regions, and with SB for pairs of ROIs involving at least one subcortical region. Figure 5a–f displays the difference in ICC scores between pairs of rs‐fMRI modalities.

## 4 DISCUSSION

To our knowledge, this study is the first to compare the test–retest reliability of rs‐fMRI metrics across single‐band and MB fMRI acquisitions, and to explore which combination of acceleration factors yields the best results and which role cortical and subcortical regions play in this context.

In the context of the ALFF measure, the regional intensity of spontaneous brain activity exhibited moderate to almost perfect ICC scores overall, with values ranging between 45.99 and 81.45 across all four rs modalities and all brain regions. This is partially in line with Golestani et al.'s (2017) results describing ALFF ICC values ranging between 65 and 85. Upon further investigation of our findings, in line with our first hypothesis, the ALFF measure was associated with higher values for MB protocols (62.13–81.45) compared with the single band sequence (49.57–68.10) for cortical regions, while it exhibited lower values for MB protocols (45.99–57.21) compared with the single band sequence (59.04–67.12) for the bilateral nuclei accumbens. It is important to note that sequences with shorter TRs (323 ms) have previously been associated with higher ALFF reliability scores compared with longer TRs (2 s; Golestani et al., 2017), however this is the first study to show a discrepancy in the reliability of spontaneous brain activity between subcortical regions and cortical regions when explicitly comparing single band and MB sequences. These findings may inform the choice of acquisition parameters for future studies exploring ALFF in the context of psychiatric disorders, where subcortical regions have particular relevance. Indeed, previous research has shown decreased ALFF in the PCC, a key node of the DMN, in bipolar disorder (Lei et al., 2017), Alzheimer disease (Zheng et al., 2019) and schizophrenia and obsessive–compulsive disorder (Zhang et al., 2021), and increased ALFF in the left AI, a core node of the salience network, in major depressive disorder with a history of childhood trauma (Wu et al., 2021). Our present findings suggest that future studies may consider using MB sequences when investigating ALFF in these cortical regions.

With regards to the seed‐to‐voxel metrics, ICC scores ranged between 9.05 and 53.56, which is in line with Golestani et al.'s (2017) findings showing ICC values ranging from 10 to 50 for seed‐based motor network connectivity. Consistent with our expectations, these scores were considerably lower compared with those for ALFF (45.99–81.45). However, a similar discrepancy in ICC scores between cortical and subcortical areas was observed across the four sequences for rs connectivity. More specifically, the ICC scores of seed‐to‐voxel analyses were higher for MB protocols (31.69–53.56) compared with the single band sequence (28.87–40.54) for cortical regions, and lower for MB protocols (9.05–21.43) compared with the single band sequence (23.40–25.86) for both subcortical regions (i.e., bilateral amygdalae and bilateral nuclei accumbens). Furthermore, for cortical regions, highest scores were obtained with MB4‐ARC1 (i.e., MB factor 4 with no in‐plane acceleration; 34.68–53.56), while MB4‐ARC2 (i.e., MB4 with an in‐plane acceleration of 2) was the MB sequence that yielded the lowest results (31.69–42.07). These findings are in line with our second hypothesis. Moreover, for subcortical regions, ICC scores decreased as the MB factor increased, with MB4‐ARC1 consistently yielding the highest MB ICC values (15.40–21.43) and MB6‐ARC1 exhibiting the lowest values (9.05–19.86). Additionally, subcortical regions consistently yielded lower ICC scores compared with cortical regions across all four rs modalities, which fits in with Shah et al.'s (2016) findings and confirms our fourth hypothesis. From a clinical perspective, it is worth noting that seed‐based analyses have been commonly used in the past to gain a better understanding of brain dysfunction in neurodevelopmental and psychiatric disorders. Our present findings revealed that future studies using MB factor 4 with no in‐plane acceleration would help build upon existing psychiatric research aiming to identify biomarkers targeting specific cortical structures. Atypical FC of the AI has previously been associated with autism spectrum disorders (Ebisch et al., 2011), while impaired FC of the medial prefrontal cortex has been proposed as a potential biomarker for alcohol dependence (Yang et al., 2021). In contrast, commonly used conventional single band sequences may be preferable for studies focusing on the FC of the nucleus accumbens, which has formerly been examined in the context of anorexia nervosa (Haynos et al., 2019) and major depressive disorder (Liu et al., 2021).

For the ROI‐to‐ROI metric, the reliability scores ranged between −3.84 and 68.46, which is lower compared with the ALFF reliability scores (45.99–81.45), as expected. Furthermore, in line with our hypotheses and the results we obtained with seed‐to‐voxel analyses, the ROI‐to‐ROI measure also showed the highest ICC values with MB4‐ARC1 for pairs of ROIs only involving two cortical regions and with single band for pairs of ROIs involving at least one subcortical region.

Taken together, these findings are strong indicators that a MB acceleration factor of 4 with no in‐plane acceleration improves the test‐reliability of rs‐fMRI metrics for cortical regions, while single band sequences are better suited for subcortical brain areas. More specifically, the lower reliability scores detected for subcortical ROIs in the context of MB protocols could reflect the lower signal to noise ratio observed in subcortical regions for MB sequences compared with single band as shown in this study. This is in line with previous studies showing higher noise amplification in subcortical regions in the context of shorter TRs (Risk et al., 2021). In particular, it has been suggested that higher sampling rates are associated with noise amplification due to individual slices being recovered from multiple simultaneously excited slices during image reconstruction (Risk et al., 2021).

Additionally, in our study, the MB protocol with the lowest total acceleration (i.e., 4 with no in‐plane acceleration) was the MB modality that yielded the best reliability results for cortical areas. These results accord with the negligible decrease in tSNR previously observed by Preibisch et al. (2015) with a total acceleration of 4 (i.e., MB factor 2 with an in‐plane acceleration of 2), which is much lower than the loss in tSNR of about 64% they obtained with a total acceleration of 8 (i.e., MB factor 4 with an in‐plane acceleration of 2). In fact, in line with Preibisch et al. (2015), our findings also revealed a negligible difference in tSNR between SB‐ASSET2 and MB4‐ARC1 (i.e., a total acceleration of 4) and a significant decrease in tSNR between SB‐ASSET2 and MB6‐ARC1 for both cortical and subcortical ROIs. However, with regards to MB4‐ARC2 (i.e., a total acceleration of 8), we found the difference in tSNR between SB‐ASSET2 and MB4‐ARC2 to be significant for subcortical areas but nonsignificant for cortical areas, which only partially aligns with Preibisch et al.'s (2015) findings. This could be because Preibisch et al. (2015) presented the results of whole‐brain analyses while we presented separate findings for cortical and subcortical areas. Because of the differences in tSNR between SB‐ASSET2 and some of the MB modalities, future studies might benefit from controlling for the effect of tSNR when exploring test–retest reliability, as it may partially explain some of the differences in ICC scores across rs‐fMRI modalities.

Furthermore, physiological and motion artifacts have been shown to affect the quality and the test–retest reliability of FC patterns as well as enhance subject specificity (Birn et al., 2014; Xifra‐Porxas et al., 2021). Indeed, head motion and cardiac and breathing variations have been shown to be linked to the variance in the fMRI signal (Power et al., 2017). As such, these could also explain some of the differences in ICC scores across the four rs‐fMRI modalities.

Finally, it must be pointed out that multiarray coils exhibit a radially dependent sensitivity which reduces towards their center and which may have influenced some of the differences in ICC reliability between cortical and subcortical regions described in this article. However, it is worth highlighting that the same coil was used for single band and MB sequences and therefore issues related to reduced coil sensitivity would only play a partial role in the results observed across our measurements.

### 4.1 Limitations

One limitation of our study is that we focused our analyses on 11 ROIs chosen a priori. Even though the choice of a small number of ROIs allowed for more in‐depth analysis of the effect of ROI location on reliability scores in comparison with whole‐brain analyses, it also means that we are not able to comment on the reliability scores for other ROIs that might be of interest for other brain diseases.

Furthermore, future studies should consider exploring the effects of in‐plane acceleration more systematically and investigating other combinations of acceleration factors that were not examined here due to time constraint, such as single band with no in‐plane acceleration and MB factor 6 with in‐plane acceleration. Indeed, this would further inform the specific impact of in‐plane acceleration on reliability scores.

It is also important to note that our current results apply to data acquired on a 3 T MR750 GE scanner, which does not allow us to generalize our findings to different field strengths or any other manufacturer. Indeed, the multivariate consistency of rs‐fMRI connectivity maps has previously been shown to decrease because of variations in scanning sites and scanner manufacturers (Badhwar et al., 2020). Future research exploring the impact of various acceleration factors across different manufacturers or field strengths would be particularly useful.

Additionally, our analyses involved using anatomically defined ROIs as opposed to specific subregions derived from areas of interest. Future studies could benefit from exploring the test–retest reliability of segmented subregions using parcellation techniques as these could further inform ROI heterogeneity as well as specific factors at play in the context of test–retest reliability.

Finally, our participants were aged between 52 and 73 and we chose to focus on this age group because this study was carried out as a methods investigation to evaluate the test–retest reliability of a neuroimaging protocol for potential future inclusion in clinical trials of aging‐associated diseases. However, this means that our findings cannot be unequivocally generalized to younger age groups. Further research is needed to explore how MB and in‐plane acceleration would affect test–retest reliability across the lifespan.

## 5 CONCLUSION

In conclusion, this study provides strong evidence that MB factor 4 with no in‐plane acceleration enhances reliability scores for cortical areas, while single band yields the best reliability values for subcortical regions. Based on these findings, we recommend MB4 with no in‐plane acceleration for whole brain analyses or analyses focusing on specific cortical regions, and single band for studies aiming to specifically explore subcortical areas.

## Supporting information


**TABLE S1.** ICC scores [lower bound of the 95% confidence interval – upper bound of the 95% confidence interval] for the ALFF measure across the 11 ROIs and the four rs‐fMRI modalities. Abbreviations: AC, anterior cingulate; ACC, anterior cingulate cortex; AI, anterior Insula; ALFF, amplitude of low‐frequency fluctuations; ICC, intraclass correlation coefficient; mPFC, medial prefrontal cortex; Nacc, nucleus accumbens; PC, posterior cingulate; PCC, posterior cingulate cortex; ROIs, regions of interest; rs‐fMRI, resting‐state functional magnetic resonance imaging

**TABLE S2.** ICC scores [lower bound of the 95% confidence interval – upper bound of the 95% confidence interval] for the seed‐to‐voxel measure across all ROIs and all rs‐fMRI modalities. Abbreviations: AC, anterior cingulate; ACC, anterior cingulate cortex; AI, anterior Insula; ALFF, amplitude of low‐frequency fluctuations; ICC, intraclass correlation coefficient; mPFC, medial prefrontal cortex; Nacc, nucleus accumbens; PC, posterior cingulate; PCC, posterior cingulate cortex; ROIs, regions of interest; rs‐fMRI, resting‐state functional magnetic resonance imaging

**FIGURE S1.** Unprocessed echo planar imaging images of slice 21 for run 1 from each of the 24 participants for (a) SB‐ASSET2; (b) MB4‐ARC1; (c) MB4‐ARC2; and (d) MB6‐ARC1; axial view.

## Data Availability

Data will be made available on reasonable request.
