# Supplementary material for: Comparing the test–retest reliability of resting‐state functional magnetic resonance imaging metrics across single band and multiband acquisitions in the context of healthy aging
Source: Hum Brain Mapp. 2022 Dec 22;44(5):1901–12. doi: 10.1002/hbm.26180 (PMC9980889; doi:10.1002/hbm.26180)
Supplement: Supplementary file 1 — TABLE S1. ICC scores [lower bound of the 95% confidence interval – upper bound of the 95% confidence interval] for the ALFF measure across the 11 ROIs and the four rs‐fMRI modalities. Abbreviations: AC, anterior cingulate; ACC, anterior cingulate cortex; AI, anterior Insula; ALFF, amplitude of low‐frequency fluctuations; ICC, intraclass correlation coefficient; mPFC, medial prefrontal cortex; Nacc, nucleus accumbens; PC, posterior cingulate; PCC, posterior cingulate cortex; ROIs, regions of interest; rs‐fMRI, resting‐state functional magnetic resonance imaging [file HBM-44-1901-s001.docx]

|  | SB-ASSET2  240 vols | MB4-ARC1  644 vols | MB4-ARC2  645 vols | MB6-ARC1  873 vols |
| --- | --- | --- | --- | --- |
| ACC (salience) | **61.29**  [60.41-62.21] | **73.34**  [72.13-74.29] | **69.92**  [68.89-70.93] | **64.25**  [63.16-65.28] |
| AC gyrus | **49.57**  [48.95-50.25] | **74.63**  [74.19-75.08] | **70.59**  [69.98-71.33] | **62.13**  [61.30-63.00] |
| AI left (salience) | **68.10**  [66.24-70.48] | **78.66**  [77.53-79.31] | **81.45**  [80.83-82.38] | **68.28**  [67.37-69.35] |
| AI right (salience) | **65.02**  [63.23-66.46] | **77.39**  [76.31-78.20] | **78.93**  [77.95-79.86] | **69.20**  [67.47-70.70] |
| Amygdala left | **58.79**  [57.35-60.71] | **73.75**  [70.31-77.05] | **70.21**  [67.67-72.65] | **67.35**  [65.24-68.70] |
| Amygdala right | **62.62**  [59.66-64.74] | **71.99**  [69.41-74.22] | **74.65**  [73.15-76.17] | **63.92**  [61.47-66.34] |
| mPFC (DMN) | **57.66**  [56.51-58.86] | **76.74**  [75.80-77.66] | **78.04**  [77.33-78.88] | **79.74**  [79.00-80.56] |
| NAcc left | **67.12**  [63.67-71.47] | **45.99**  [42.47-50.90] | **57.21**  [55.48-59.66] | **51.41**  [48.89-53.97] |
| NAcc right | **59.04**  [57.15-64.59] | **52.20**  [50.78-54.22] | **50.83**  [49.53-52.92] | **50.40**  [45.08-54.30] |
| PCC (DMN) | **67.48**  [66.98-68.02] | **79.47**  [79.16-79.80] | **75.05**  [74.56-75.37] | **73.71**  [73.24-74.09] |
| PC gyrus | **55.50**  [54.68-56.36] | **77.37**  [76.63-78.04] | **72.88**  [72.17-73.42] | **65.01**  [64.35-65.68] |

Appendix Table 1. ICC scores [lower bound of the 95% confidence interval – upper bound of the 95% confidence interval] for the ALFF measure across the eleven ROIs and the four rs-fMRI modalities.
