# Supplementary material for: Comparing the test–retest reliability of resting‐state functional magnetic resonance imaging metrics across single band and multiband acquisitions in the context of healthy aging
Source: Hum Brain Mapp. 2022 Dec 22;44(5):1901–12. doi: 10.1002/hbm.26180 (PMC9980889; doi:10.1002/hbm.26180)
Supplement: Supplementary file 2 — TABLE S2. ICC scores [lower bound of the 95% confidence interval – upper bound of the 95% confidence interval] for the seed‐to‐voxel measure across all ROIs and all rs‐fMRI modalities. Abbreviations: AC, anterior cingulate; ACC, anterior cingulate cortex; AI, anterior Insula; ALFF, amplitude of low‐frequency fluctuations; ICC, intraclass correlation coefficient; mPFC, medial prefrontal cortex; Nacc, nucleus accumbens; PC, posterior cingulate; PCC, posterior cingulate cortex; ROIs, regions of interest; rs‐fMRI, resting‐state functional magnetic resonance imaging [file HBM-44-1901-s003.docx]

|  | SB-ASSET2  244 vols | MB4-ARC1  644 vols | MB4-ARC2  645 vols | MB6-ARC1  873 vols |
| --- | --- | --- | --- | --- |
| ACC (salience) | **37.51**  [37.30-37.72] | **38.14**  [37.81-38.41] | **33.21**  [32.94-33.47] | **38.45**  [38.20-38.71] |
| AC gyrus | **28.87**  [28.65-29.11] | **34.68**  [34.47-34.90] | **31.69**  [31.44-31.94] | **32.01**  [31.77-32.23] |
| AI left (salience) | **38.96**  [38.72-39.20] | **47.20**  [46.96-47.46] | **35.14**  [34.85-35.40] | **43.47**  [43.23-43.71] |
| AI right (salience) | **40.54**  [40.30-40.79] | **47.37**  [47.04-47.64] | **37.62**  [37.33-37.94] | **48.04**  [47.79-48.29] |
| Amygdala left | **25.51**  [25.25-25.80] | **20.45**  [20.19-20.73] | **18.97**  [18.69-19.22] | **19.86**  [19.54-20.21] |
| Amygdala right | **23.40**  [23.14-23.72] | **21.43**  [21.20-21.67] | **19.38**  [19.15-19.65] | **19.18**  [18.84-19.57] |
| mPFC (DMN) | **38.21**  [37.97-38.51] | **46.69**  [46.38-47.05] | **41.89**  [41.60-42.19] | **44.10**  [43.85-44.35] |
| NAcc left | **25.55**  [25.29-25.85] | **15.40**  [15.15-15.68] | **13.53**  [13.07-13.90] | **10.17**  [9.74-10.58] |
| NAcc right | **25.86**  [25.56-26.17] | **16.72**  [16.42-17.00] | **13.06**  [12.69-13.48] | **9.05**  [8.62-9.53] |
| PCC (DMN) | **40.34**  [40.07-40.61] | **53.56**  [53.32-53.83] | **42.07**  [41.82-42.37] | **43.40**  [43.10-43.68] |
| PCC gyrus | **37.48**  [37.25-37.71] | **44.38**  [44.10-44.65] | **34.95**  [34.73-35.23] | **39.52**  [39.29-39.72] |

Appendix Table 2. ICC scores [lower bound of the 95% confidence interval – upper bound of the 95% confidence interval] for the seed-to-voxel measure across all ROIs and all rs-fMRI modalities.
