# Supplementary figures and images for: Comparing the test–retest reliability of resting‐state functional magnetic resonance imaging metrics across single band and multiband acquisitions in the context of healthy aging
Source: Hum Brain Mapp. 2022 Dec 22;44(5):1901–12. doi: 10.1002/hbm.26180 (PMC9980889; doi:10.1002/hbm.26180)

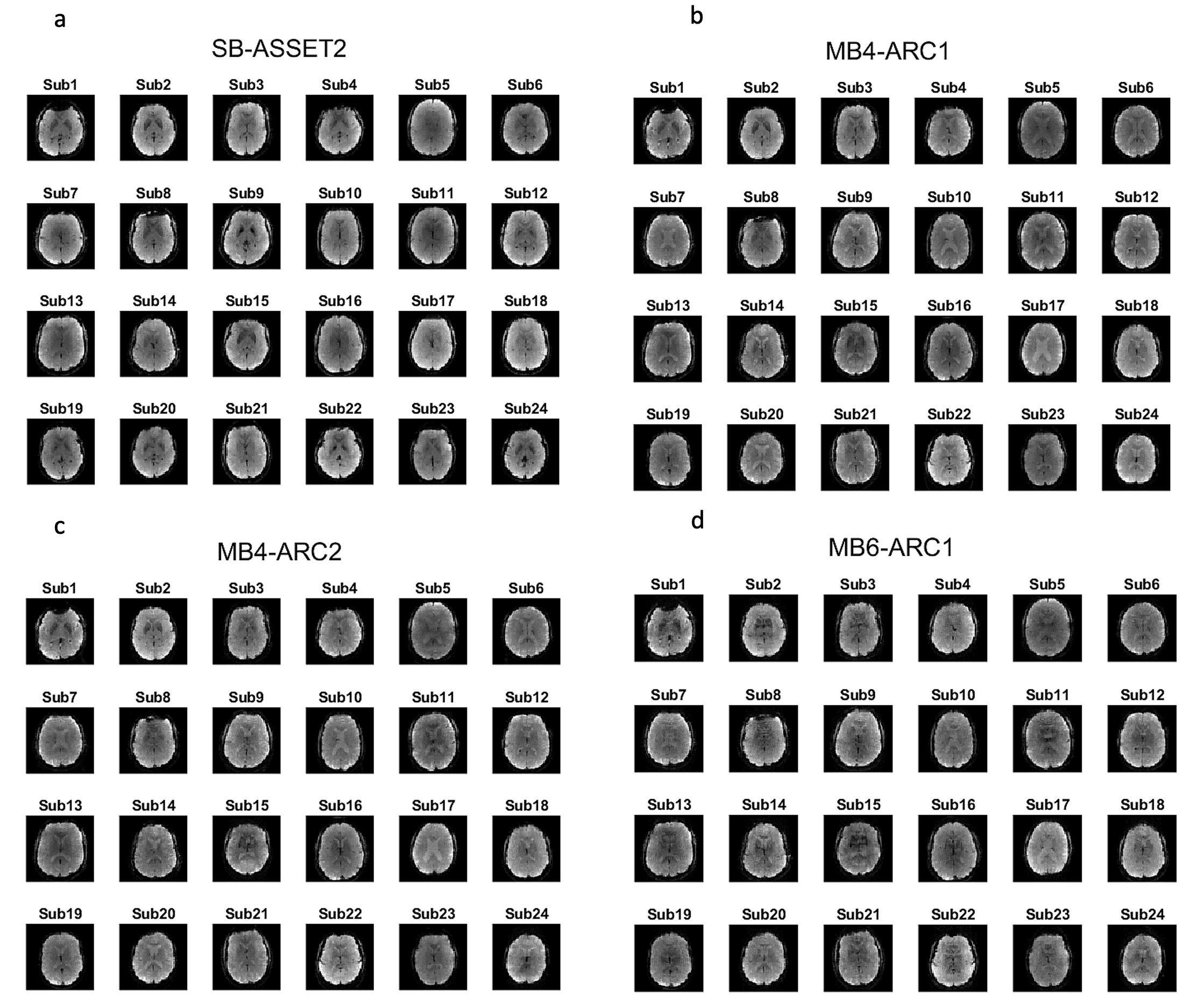

Supplement: Supplementary file 3 — FIGURE S1. Unprocessed echo planar imaging images of slice 21 for run 1 from each of the 24 participants for (a) SB‐ASSET2; (b) MB4‐ARC1; (c) MB4‐ARC2; and (d) MB6‐ARC1; axial view. [file HBM-44-1901-s002.tif]
